# School Violence and Teacher Professional Engagement: A Cross-National Study

**DOI:** 10.3389/fpsyg.2021.628809

**Published:** 2021-04-15

**Authors:** Youcai Yang, Lixia Qin, Ling Ning

**Affiliations:** ^1^Key Laboratory of Adolescent Health Assessment and Exercise Intervention of Ministry of Education, East China Normal University, Shanghai, China; ^2^College of Physical Education and Health, East China Normal University, Shanghai, China; ^3^University of Wisconsin System, Madison, WI, United States; ^4^Office of Assessment and Planning, University of Colorado Boulder, Boulder, CO, United States

**Keywords:** school violence, teacher professional engagement, structural equation modeling (SEM), cross-national comparison, teacher self-efficacy, school climate

## Abstract

School violence research has mainly focused on the impact on students. Very few studies, even fewer from a cross-cultural perspective, have examined the relationships between school violence and teacher professional engagement, and the role played by teacher self-efficacy and school climate related factors. The present study utilizes a SEM research methodology to analyze the 2013 TALIS data. The purpose is to understand and compare the relationships in four different cultural contexts; the U.S., England, South Korea, and Mexico. Results indicate, on average, that the significant and negative impacts of school violence on teacher professional engagement are partly mediated by teacher self-efficacy. The negativity of school violence is significantly alleviated by enhancing participation among school stakeholders and improving teacher–student relationships. The relationships among the factors apply across all four cultural systems, though, the effects of factors and variables vary to a degree. The paper also discusses other relevant issues and differences as well as their implications.

## Introduction

School violence is a social issue that has drawn significant attention and debate among different parties in the U.S. On March 14, 2018, in response to the Marjory Stoneman Douglas High School massacre in Florida, the U.S. House of Representatives voted overwhelmingly in support of legislation aimed at reducing school violence.

Ranging from aggression against school property, verbal abuse of students or teachers, physical bullying, to lethal rampages, school violence has a traumatic impact on all members of a school community (Lester et al., 2017). However, while most research on school violence to date has focused on students (e.g., Robers et al., 2010; Cornell, 2017), the effect of school violence on teachers has received little media, research, and policy attention within the U.S. and across the world (Espelage et al., 2013). Research examining how school violence impacts teachers' well-being, job satisfaction, and future career decisions has been very limited. More importantly, very little research has addressed how the effects of school violence on teachers can vary in conjunction with school-related and culture-related contextual factors (e.g., Galand et al., 2007; McMahon et al., 2017).

Research has shown that violence directed toward teachers has reached concerning levels, requiring further investigation (e.g., Tiesman et al., 2013; Martinez et al., 2015). For instance, in a nationwide survey in the U.S., researchers reported that 80% of teachers experienced at least one of 11 different forms of school violence during the current or past school year (McMahon et al., 2014). The International Survey on Teaching and Learning (TALIS, 2013) shows that the U.S. ranks highest in the category of school violence. Around 14% of principals reported that their teachers have suffered some type of violence from students, such as intimidation or verbal abuse, which is greater than the international average of 10% (OECD, 2014). Another study indicated that the victimization rate of teachers varied by state and, more specifically, by the rate of teachers who had been threatened with physical violence; in some states, the rate was as high as 17% (e.g., Zhang et al., 2016).

A growing body of literature from different developed and developing countries demonstrates that school violence is associated with teacher's disengagement, turnover, and some other negative consequences, such as their emotional wellbeing. Yet the prevalence of school violence and its impact on teachers' professional engagement is not the same between countries. [see e.g., Mexico (Estévez et al., 2016), South Korea (Moon et al., 2015), Canada (Berg and Cornell, 2016), Malaysia (Santos and Tin, 2018)]. Almost no studies have comparatively examined the prevalence, relevance, or the variety of school violence that occurs in different international contexts, and even fewer have studied the factors associated with school violence that can impact the professional engagement of teachers in the U.S. and in other contexts worldwide (Akiba et al., 2002; Baker et al., 2005).

Researchers have warned that an over emphasis on intra country studies may cause insularity that could potentially lead to insensitivity of educational policies to various situations (Dolton and Marcenaro-Gutierrez, 2011). The current cross-country study was designed to investigate the impact of school violence on teacher professional engagement and to compare the variation of teacher responses to violence in different institutional and national settings. The purpose is to contribute to the development of generalizable theories, policies, and intervention strategies that can mitigate the impact of school violence on teachers across countries.

Specifically, this multiple-country study examined the extent to which school violence influenced teachers' professional engagement and the mediation role played by teacher self-efficacy. It further explored how the school climate related factors, such as participation among stakeholders and teacher–student relationships, contribute to reducing school-based violence across countries. Four countries were selected for this study: the U.S., England, South Korea (or the Republic of Korea), and Mexico. The four countries, located in three continents, are diverse across several dimensions including geographical features, economic indicators, cultural characteristics, and educational systems (OECD, 2014). Given that research on school violence and teacher professional engagement has predominantly been conducted in the U.S., the U.S. was included to situate the findings. England, which has somewhat similar sociocultural and educational systems to those in the U.S., was chosen to examine the replicability of the findings from the U.S. in a completely different set of teacher samples from England. South Korea was chosen to represent the geographical and sociocultural profile of East Asia (Confucian, Collectivism; Hofstede and Hofstede, 2005). And Mexico was chosen to represent the Latin American context characterized by collectivism and interdependence (Estévez et al., 2016). Three research questions guided the present study:

1. What is the effect of school violence on teachers' professional engagement in the U.S., England, South Korea, and Mexico?
2. How does teacher self-efficacy mediate the effects of school violence on teacher professional engagement in each of the four countries?
3. Does participation among stakeholders and teacher–student relationships contribute to prevention of school violence?

## Conceptual Model

### Impact of School Violence on Teacher Professional Engagement

The literature suggests that school violence in K-12 public school settings is a common, worldwide problem that has been found to be associated with serious adverse consequences on teacher professional engagement (e.g., work-related stress and burnout, decreased teaching effectiveness, disengagement from teaching, and turnover; Ingersoll, 2001; Pas et al., 2010; Martinez et al., 2015; Wang et al., 2015). In fact, a lack of safety can hamper teachers' ability to “deal completely with the demands of the job” (Schaufeli et al., 2002, p. 73) and can eventually worsen teacher professional disengagement (Boyd et al., 2011). Empirical evidence in the U.S. has documented that perceptions of student behavior and school safety are among the strongest predictors of teacher career decisions (Ingersoll, 2002; Marinell and Coca, 2013; Kemper, 2017). In addition, Newman et al. (2004) reported that teachers who worry about their safety are more likely to leave the teaching profession altogether.

Teachers' feelings toward school violence can have significant implications for the educational system, affecting the quality and continuity of children's education through the way teachers teach, through their absentee rate, and through their relationship with their students (Payne et al., 2003; Finley, 2004; DeVoe et al., 2005). Other researchers have shown that teachers who felt unsafe at school due to potential violence had lower levels of job satisfaction (Williams et al., 1989), tended to be unmotivated and less committed to their job (Van Ginkel, 1987; Vettenburg, 2002), and even left the profession altogether (Ingersoll, 2001; Scheckner et al., 2002).

### School Climate

A range of previous studies indicate that teachers' perceptions and experiences of school violence have been directly and indirectly affected by school contexts, an ecosystem of multiple social environments which defines school climate (see e.g., Karcher, 2002). The joint efforts between schools, parents, and communities helps buffer and prevent the incurrence of school violence. Researchers have indicated that parents' involvement in school-based activities and community support and engagement in school-wide violence prevention efforts allow schools and teachers to nurture and maintain proper behaviors. The active engagement of parents and strong community support play important and positive mediating roles leading to positive student outcomes, reduced teachers' subjectivity to inappropriate school-based behaviors, and decreased school violence (Sampson et al., 1997; Welsh, 2000; Benbenisty and Astor, 2005; Ricketts, 2007; Gage et al., 2014). Involving parents and community members in meaningful ways allow schools to maintain appropriate behaviors (Gage et al., 2014).

The definition of factors of an ecosystem in a school social environment (aka the constructs of school climate) varies across different studies (De Pedro et al., 2016; Wang and Degol, 2016). One of the important factors consistently identified across research is the community in which the quality of interactions and relationship among school members is assessed (Wang and Degol, 2016). In the current study, we selected two aspects of school climate that are theoretically and empirically very important: teachers' self-report regarding the participation among stakeholders and student-teacher relationships. Research indicates that participation among stakeholders promotes a supportive educational environment by establishing mutual goals between the school, family, and community (Christenson, 1995). Positive relationships establish trust and respect between students and teachers (Hopson and Lee, 2011) and build students' sense of attachment and bonding to school (Kotok et al., 2016).

### Teacher Self-Efficacy

Teacher self-efficacy was found to positively predict teachers' psychological well-being and negatively predict their intentions to quit (Wang et al., 2015). Based on Albert Bandura's self-efficacy theory (Bandura, 1986), teacher efficacy is a situation-specific construct meaning teacher's self-efficacy varies across different situations or contexts. When teachers feel they are not capable of controlling and managing teaching situations, a sense of powerlessness may result in lower self-efficacy (Rosenholtz, 1989). Therefore, positive experiences become an encouraging reward while negative experiences may result in disengagement or encourage leaving the profession (Ware and Kitsantas, 2011). Besides the contextual factors, this study also added teacher self-efficacy as an impacting factor that related to teachers' perception on their teaching and school. Research has indicated the correlations between teacher self-efficacy and teacher burnout and attrition (e.g., Schwarzer and Hallum, 2008). Teachers' self-efficacy plays important roles in navigating the impact of school violence and reducing the effects that has on their professional engagement.

Among the factors influencing teachers' self-efficacy, student behavior problems have become the next most cited factor relating to lower teacher self-efficacy and their decisions to quit (e.g., Borman and Dowling, 2008; Brill and McCartney, 2008). Substantial disappointment has been observed among teachers who are experiencing overwhelming student discipline problems, which lead to teachers questioning their teaching ability and their professional choices. Student misbehaviors might make teachers feel less able to carry out their teaching tasks. The issue is even more significant among beginner teachers who grapple with higher levels of pressure regarding their relationship with students and their ability to manage student behavior (e.g., Lukens et al., 2004). Friedman (1995) revealed that 22% of the variance in predicting teacher burnout can be explained by typical student misbehaviors.

### Teacher and School Characteristics

The effects of school violence on teachers differ by teacher characteristics. Some studies have found that male teachers are more likely to be affected than their female colleagues (Robers et al., 2013), whereas other studies suggest that female teachers are more vulnerable to violence (e.g., Wei et al., 2013). Using the survey responses of 4,371 Minnesota educators, the a uthors found that female and non-white teachers tended to experience more school violence, while experienced teachers were less likely to be affected by school violence (Wei et al., 2013). Differences may be partially due to the type of violence reported (McMahon et al., 2014).

Regarding school characteristics, a large number of studies have found that school violence in larger schools in urban areas is more prevalent than in other schools (Stewart, 2003). School size may also be related to safety, although findings have been inconsistent (Klein and Cornell, 2010; Robers et al., 2013). School poverty has been found to be associated with school violence. Teachers report less safety in higher poverty schools (Steinberg et al., 2013). Poverty explained nearly 20% of differences in teacher reports of safety.

**Figure 1:**
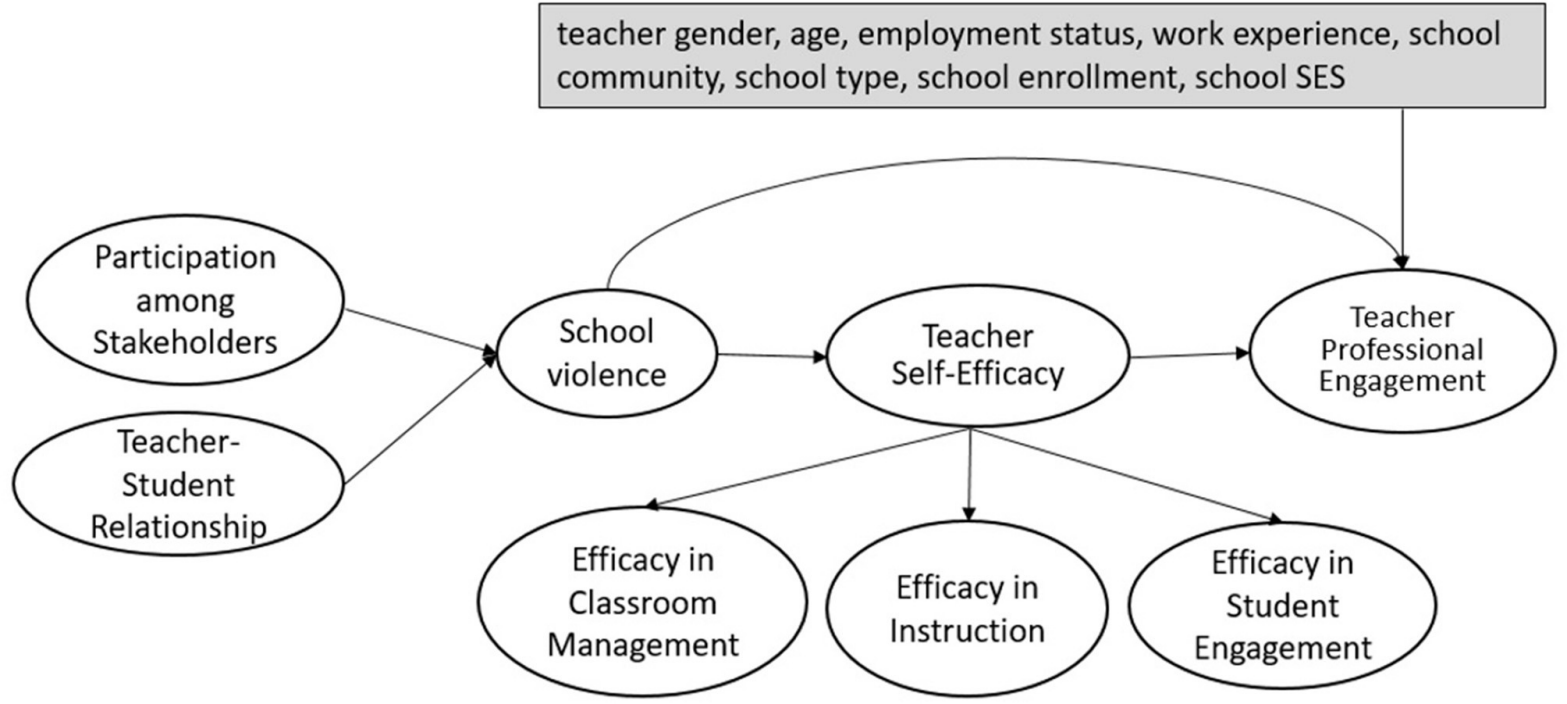
SEM model on school violence and teacher professional engagement.

The present study builds upon previous research by testing how the perceived school violence (exogenous latent variables) affects teachers' professional engagement (endogenous latent variable). Besides the direct effect aforementioned in previous research, the model also investigates whether the effect of school violence on professional disengagement is mediated by teacher self-efficacy (Figure 1), whether the same model holds true across four countries, and how the effects differ among different cultural contexts. It is hypothesized that school violence affects teacher professional engagement but is mediated by teacher self-efficacy (see Figure 1). We, therefore, not only test the direct relationship between school violence and teachers' professional engagement, but also test the mediating effect of teachers' self-efficacy on these relationships. We also identify participation among stakeholders and teacher–student relationships, which may address the level of school violence.

## Methods

### Data and Samples

The dataset used in the study was the 2013 Teaching and Learning International Survey (TALIS) administered by the Organization for Economic Co-operation and Development (OECD). This international large-scale survey of teachers, principals, teaching, and school environment closely looked at the school and classroom features that influenced teacher effectiveness. In general, the TALIS 2013 instruments include selected antecedent variables, school inputs, processes and a limited set of outcomes.

In order to ensure that a representative sample of the target population was tested in each participating country, TALIS 2013 used a two-stage stratified probability sampling design. A school was excluded if the response rate was lower than 50% of sampled teachers. Both paper-and-pencil and online survey administration modes were used (OECD, 2014). The sampling weights were applied at the teacher level to reduce the sampling error caused by unequal probability of selection (OECD, 2014).

In total, the study sample includes responses from 10,316 teachers (grades 9 and 10) at the second stage and from 638 schools at the first stage. More specifically, the samples of four countries contain responses from 1,857 teachers in 122 schools in the U.S., 2,825 teachers in 177 schools in South Korea, 2,496 teachers in 152 schools in England, and 3,138 teachers in 187 schools in Mexico. The TALIS data provide detailed descriptions of teacher and school characteristics (OECD, 2014).

### Variables

#### Independent Variable

In the TALIS data, school violence measures school vandalism and theft, intimidation or verbal abuse among students (or other forms of non-physical bullying), physical injury caused by violence among students, intimidation or verbal abuse of teachers, and use/possession of drugs and/or alcohol. One sample item is “Intimidation or verbal abuse of teachers or staff.” All items in this construct were answered on a five-point scale. Response categories were 1 for never, 2 for rarely, 3 for monthly, 4 for weekly, and 5 for daily.

Participation among stakeholders, measures whether stakeholders such as staff, parents, or guardians and students have opportunities to participate in school decisions and to what extent the school has a culture of shared responsibility and mutual support regarding school issues. One sample item is “This school provides parents with opportunities to participate in school decisions.” All items were answered on a four-point scale, with response categories of 1 for strongly disagree, 2 for disagree, 3 for agree and 4 for strongly agree. Teacher–student relationship (four items) measures whether teachers and students get along in the school and to what extent teachers care about students' well-being and respect their opinions. One sample item is “Most teachers in this school are interested in what students have to say.” The measures of teacher–student relations have the same scale points and response categories as the measures of participation among stakeholders.

#### Mediating Variable

The teacher self-efficacy scale measures teachers' efficacy in classroom management, efficacy in instruction and efficacy in student engagement. Each sub-scale contains four items on a four-point scale with response categories of 1 for not at all, 2 for to some extent, 3 for quite a bit, and 4 for a lot. One sample item that measures teacher efficacy in classroom management is “To what extent can you calm a student who is disruptive or noisy?”

#### Dependent Variable

Teacher professional engagement measures to what extent teachers are engaged in their teaching profession. Four items have been included, all items were measured on a four-point scale, for which the response categories were 1 for strongly disagree, 2 for disagree, 3 for agree, and 4 for strongly agree. One sample item that measures teachers' professional engagement is “If I could decide again, I would still choose to work as a teacher.”

#### Control Variable

Several control variables were included in the final SEM model (see Figure 1) to remove their potential effects on the relationship between school violence and teacher professional engagement. Prior research has highlighted their importance in explaining the variations in teacher professional engagement (see e.g., Torres, 2019). The control variables are teachers' demographic variables [i.e., gender (TT2G01), age (TT2G02), school-related work experience (TT2G05A), employment status (TT2G03)] and schools' contextual variables [i.e., school type (TC2G10), school community (TC2G09), school enrollment (TC2G14), and percentage of students from socioeconomically disadvantaged homes (TC2G15C)]. Some variables were recoded to simplify the analytical process and also to remove the small sample effect of some categories in certain control variables. Specifically, employment status (TT2G03) was recoded into full-time and non-full time (the reference category). School community (TC2G09) was combined into three categories of Rural/Village, Small Town/Town (the reference category), or City/Large City. Percentages of students from socioeconomically disadvantaged homes (TC2G15C)] were grouped into two categories of schools having over 30% students or below 30% students from socioeconomically disadvantaged homes (the reference category).

### Analytic Procedures

The average percentage of missingness of the sample data used in the present analyses, across all four countries, ranges from 2.8 to 4.6%. Prior to the analyses, listwise deletion was used to remove cases with missing data. In each analysis, weights were adjusted at the teacher level to account for the response errors due to unequal probability of sample selection.

TALIS (2013) was collected using stratified multi-stage sampling methods, with teachers nested within schools, which in turn were nested within countries. The clustered data from a complex survey usually require multi-level analyses. We calculated the design effect of each item measuring teachers' professional engagement and teachers' self-efficacy in each country [i.e., *Design Effect* = 1+(*Average Cluster Size*−1) × *Intraclass Correlation* ], where the average cluster size is the average number of teachers across all schools per country and the Intraclass Correlation (ICC) explains the average shared variance across teachers within a given school in a country, controlling for individual teacher variation. The results indicate that the design effect of the items for each of the two outcomes across the four countries, ranges from 1.58 to 1.89, which is below the conventional cut-off of 2 (Maas and Hox, 2004). Given the very large cluster size in each school (Lai and Kwok, 2015), it is safe to assume that the responses to the outcomes from the teachers are relatively independent and the application of a single-level analysis did not lead to overly biased results. The small ICC (ranging from 0.01 to 0.06 for items measuring teacher professional engagement across four countries) found with the sample implies that there is a very low variability between-schools in terms of teachers' engagement and self-efficacy. At the same time, however, the within-school variability in teachers' professional engagement or self-efficacy can be high, which is the focus of the present research study.

Structural equation modeling (SEM) approaches were used to test the hypotheses. Specifically, the weighted least square mean and variance adjusted (WLSMV) estimation method in Mplus 8 (Muthén and Muthén, 1998–2017) was used. The WLSMV estimator was chosen because it was designed to model ordered or categorical data. Different from its competing estimators such as Maximum Likelihood Robust (MLR), which assumes the outcomes to be continuous, the specification of outcomes to be categorical using WLSMV leads to non-linear models that are robust to assumptions of multivariate normality and non-independence observations (Brown, 2006; Li, 2016). The model (see Figure 1) was analyzed following the two-step modeling guidelines recommended by Anderson and Gerbing (1988). The first step involved examining the validity of the measurement component using Confirmatory Factor Analyses (CFA). The second step estimated the fully latent structural regression model among the latent constructs given that the measurement models fit the data adequately.

The present study used multiple fit indices to assess the adequacy of model fit to the data and the comparison of competing models. The commonly used fit indices to determine the adequacy of SEM models are root-mean-square error of approximation (RMSEA, Steiger, 1990), the Comparative Fit Index (CFI, Bentler, 1990), and the Tucker-Lewis Index (TLI, Tucker and Lewis, 1973). The cutoff criteria for the four commonly used fit indices are: both CFI and TLI are acceptable if above 0.90; RMSEA is acceptable if below 0.08. χ^2^ test statistics were also reported but not relied on for model comparison due to the undesirable performance of the χ^2^ test statistic; studies have shown that the incidental sample characteristics (e.g., skewed distributions, large sample size) may lead to an inflated χ^2^ test statistic (see e.g., Saris et al., 2009; Ning and Luo, 2017). Modification index (MI) or Lagrange multiplier was also used to assess model fit improvement. Generally speaking, a MI value greater than a critical χ^2^ value of 3.84 (given *df* =1 and α = 0.05) suggests an appreciable improvement in model fit if the model were modified to freely estimate that particular parameter, given that the *post-hoc* modification is theoretically justifiable.

The condition of partial *measurement invariance* was implemented throughout the factor structure assessment process in this study. Contrary to full measurement invariance, which requires all the measurement parameters of all items to be identical across all countries, partial measurement invariance allows a subset of measurement parameters to function differentially across countries, recognizing that in across-cultural/national studies, some items in a measuring instrument may operate in a way that is very specific to a country (see Bentler, 2005). Modification indexes were used here to assess the improvement of the model fit to the data in each country, allowing for the deletion and cross-loading of items, given that the *post-hoc* modification is theoretically justified. The multiple group analyses of each model showed that a weak configural invariance across all four countries and the overall factor structure holds similarly for all four countries.

## Results

Tables 1, 2 summarize the demographic information on the participating teachers and schools in each of the countries, with complete cases for the variables used in the analyses. Table 3 presents the descriptive statistics of TALIS indicators for each scale used in the present study. The reliability coefficient alphas for the scales range from 0.60 to 0.86 across all four countries and are detailed in **Table 6**. Interested readers can retrieve the TALIS 2013 technical report for the further description of the reliability coefficient alphas for each scale for all countries (OECD, 2014). Before assessing the 7-factor model (M1), the construct validity of each single-factor structure was assessed separately for each country through confirmatory factor analysis (CFA) using Mplus 8.0. The examination of the model fit indices showed that the CFA for each single-factor structure fits the data for each country adequately. To save space, the tabulation of the CFA model fit indices for the validity of each individual construct, in each country, is not presented. Interested readers can contact the third author for details of M1 estimations.

**Table 1:** Demographic information of participatory teachers for each country.

**Variables (TALIS Index)**	**Frequency (%) or Mean (SD)**			
**Teacher Level**	**USA (*N* = 1,857)**	**KOR (*N* = 2,825)**	**ENG (*N* = 2,496)**	**MEX (*N* = 3,138)**
Gender (TT2G01)				
Female	1,020 (67.4%)	1,758 (69.2%)	1,222 (63.8%)	413 (47.9%)
Age (TT2G02)	42.0 (11.39)	42.5 (9.17)	39.0 (10.19)	39.3 (10.1)
Employment Status (TT2G03)				
Full time	1,463 (96.7%)	2,507 (99.3%)	1,660 (86.7%)	303 (35.2%)
School related work experience (TT2G05A)	8.53 (7.49)	3.90 (5.89)	7.77 (7.10)	8.25 (7.72)

**Table 2:** Demographic information of participatory schools for each country.

**Variables (TALIS Index)**	**Frequency (%) or Mean (SD)**			
**School/Principal Level**	**USA (*N* = 122)**	**KOR (*N* = 177)**	**ENG (*N* = 152)**	**MEX (*N* = 187)**
School community (TC2G09)				
Rural/Village	15 (15.3%)	19 (11.9%)	6 (4.1%)	19 (10.6%)
(Small) Town	46 (47.0%)	15 (9.3%)	88 (61.1%)	50 (27.7%)
(Large) City	37 (37.7%	126 (78.8%)	50 (34.8%)	111 (61.7%)
School Type (TC2G10)				
Public	89 (90.8%)	133 (83.2)%	79 (54.9%)	140 (77.78%)
Private	9 (9.1%)	27 (16.9%)	65 (45.1%)	40 (22.2%)
School Enrollment (TC2G14)	795.6 (580.0)	789.8 (378.8)	1,073 (376.9)	567.4 (469.5)
Students from socioeconomically disadvantaged homes (TC2G15C)				
30% or Below	29 (29.6 %)	147 (91.2%)	110 (76.4%)	104 (57.7%)
31% or Above	69 (70.4%)	14 (8.8%)	43 (23.6%)	76 (42.3%)

**Table 3:** Descriptive statistics of TALIS indices used in the study.

**Construct**	**Item (TALIS Index)**	**Mean (SD)**				**Construct**	**Item (TALIS Index)**	**Mean (SD)**			
		**USA**	**KOR**	**ENG**	**MEX**			**USA**	**KOR**	**ENG**	**MEX**
Teacher professional engagement	The advantages of being a teacher clearly outweigh the disadvantages (TT2G46A)	2.5 (0.72)	3.05 (0.63)	3.11 (0.74)	3.05 (0.83)	School violence	Vandalism and theft (TC2G32D)	2.24 (0.64)	2.15 (0.62)	2.04 (0.41)	2.22 (1.04)
	If I could decide again, I would still choose to work as a teacher (TT2G46B)	3.18 (0.78)	2.77 (0.82)	3.06 (0.81)	3.59 (0.61)		Intimidation or verbal abuse among students (or other forms of non-physical bullying) (TC2G32E)	2.95 (0.89)	2.4 (0.73)	2.5 (0.87)	2.76 (1.17)
	I regret that I decided to become a teacher (TT2G46D)	1.54 (0.65)	1.98 (0.73)	1.60 (0.71)	1.23 (0.56)		Physical injury caused by violence among students (TC2G32F)	2 (0.54)	2.11 (0.61)	1.99 (0.49)	2.21 (0.91)
	I wonder whether it would have been better to choose another profession (TT2G46F)	2.09 (0.92)	2.3 (0.80)	2.12 (0.90)	1.59 (0.77)		Intimidation or verbal abuse of teachers or staff (TC2G32G)	1.99 (0.80)	1.5 (0.54)	2.08 (0.74)	1.71 (0.75)
Efficacy in classroom management	To what extend can you control disruptive behavior in the classroom (TT2G34D)	3.37 (0.77)	2.96 (0.69)	3.39 (0.69)	3.27 (0.67)	Efficacy in student engagement	To what extend can you get students to believe they can do well in school work (TT2G34A)	3.26 (0.77)	3 (0.66)	3.5 (0.63)	3.31 (0.67)
	To what extend can you make my expectations about student behavior clear (TT2G34F)	3.61 (0.64)	2.84 (0.66)	3.67 (0.55)	3.24 (0.67)		To what extend can you help my students value learning (TT2G34B)	3.15 (0.88)	3.01 (0.67)	3.34 (0.7)	3.35 (0.69)
	To what extend can you get students to follow classroom rules (TT2G34H)	3.41 (0.75)	3.01 (0.66)	3.50 (0.62)	3.24 (0.67)		To what extend can you motivate students who show low interest in school work (TT2G34E)	2.88 (0.85)	2.70 (0.71)	3.05 (0.74)	3.04 (0.84)
	To what extend can you calm a student who is disruptive or noisy (TT2G4I)	3.24 (0.81)	2.91 (0.70)	3.29 (0.71)	3.1 (0.73)		To what extend can you help students think critically (TT2G34G)	3.18 (0.76)	2.74 (0.69)	3.14 (0.7)	3.31 (0.67)
Efficacy in instruction	To what extend can you craft good questions for my students (TT2G34C)	3.32 (0.75)	2.961 (0.65)	3.39 (0.66)	3.22 (0.67)	Teacher-student relationship	Teachers get along well with each other (TT2G45A)	3.19 (0.60)	3.14 (0.49)	3.37 (0.55)	3.09 (0.65)
	To what extend can you use a variety of assessment strategies (TT2G34J)	3.27 (0.80)	2.78 (0.67)	3.38 (0.64)	3.15 (0.7)		Most teachers in this school believe that the students' well-being is important (TT2G45B)	3.50 (0.58)	3.11 (0.54)	3.37 (0.55)	3.34 (0.64)
	To what extend can you provide an alternative explanation (TT2G34K)	3.53 (0.71)	3.05 (0.65)	3.6 (0.55)	3.46 (0.58)		Most teachers in this school are interested in what students have to say (TT2G45C)	3.26 (0.65)	3.10 (0.51)	3.37 (0.55)	3 (0.69)
	To what extend can you implement alternative instructional strategies (TT2G34L)	3.28 (0.82)	2.73 (0.70)	3.23 (0.71)	3.24 (0.68)		If a student from this school needs extra assistance, the school provides it (TT2G45D)	3.34 (0.68)	2.86 (0.65)	3.45 (0.58)	2.81 (0.83)
Participation among stakeholders	Staff active participate in school decisions (TT2G44A)	2.67 (0.81)	2.62 (0.73)	2.54 (0.78)	2.47 (0.92)	Participation among stakeholders	A culture of shared responsibility (TT2G44D)	2.67 (0.84)	2.72 (0.65)	2.66 (0.73)	2.79 (0.84)
	parents active participate in school decisions (TT2G44B)	2.77 (0.76)	2.87 (0.59)	2.72 (0.65)	2.64 (0.82)		collaborative culture by mutual support (TT2G44E)	2.73 (0.84)	2.76 (0.67)	2.69 (0.77)	2.67 (0.86)
	Students active participate in school decisions (TT2G44C)	2.55 (0.85)	2.69 (0.66)	2.85 (0.63)	2.35 (0.83)						

The model fit information of four models (M1–M3) is summarized in Table 4. The first model (M1) tested was the 7-factor CFA model that specified the relations of indicator variables to underlying constructs, while allowing the inter correlation of the constructs to be freely estimated. Modification indices were carefully examined for parameters that contribute most substantially to model misfit. Cross-loadings of some items on certain subscales were found to contribute substantially to the model's misfitting of the data in some countries. For example, item TT2G34G (“Help students think critically”), which was designed to measure the subscale of teacher self-efficacy in student engagement, was also found to measure the subscale of efficacy in instruction in the data from the U.S., South Korea, and England, but not in the data from Mexico. A re-specified *post-hoc* model that allows free estimation of the cross-loadings of certain items on a subscale to which they were not assigned, yielded significantly improved model fit statistics. The model fit indices indicate that the final M1 had an optimal fit to the data across the four countries. The values of CFI and TLI lie in the range of 0.96–0.98 with RMSEA being around 0.04 among the four countries. The adequacy of the 7-factor model provided a well-fitting baseline structure on which to build the following more complex models.

**Table 4:** Evaluating model fit adequacy.

**Model**	**Country**	**Model Fit Indices**			
		**χ^2^ (*df*)**	**CFI**	**TLI**	**RMSEA**
The 7-factor CFA (M1)	USA	1,597.712 (443)	0.975	0.972	0.037
	Korea	2,718.642 (506)	0.981	0.979	0.039
	England	2,187.013 (504)	0.968	0.965	0.037
	Mexico	2,638.962 (433)	0.968	0.963	0.04
The 7-factor +2nd factor CFA (M2)	USA	1,590.891 (454)	0.976	0.974	0.036
	Korea	2,863.653 (517)	0.98	0.979	0.039
	England	2,330.184 (515)	0.966	0.963	0.038
	Mexico	4,031.193 (507)	0.946	0.94	0.047
The full SEM Research Model (M3)	USA	2,437.555 (617)	0.954	0.950	0.043
	Korea	6,090.946 (617)	0.952	0.948	0.058
	England	4,481.908 (617)	0.936	0.931	0.050
	Mexico	4,754.658 (617)	0.946	0.941	0.051

M1 was then extended to a second-order CFA model (M2) accounting for the theoretical constructs proposed by OECD where the overall teacher self-efficacy scale is measured by three sub-domains: classroom management, instruction, and student management. The final model fit of M2, as presented in Table 4, shows that M2 fits the data from each of the countries adequately, with the values of CFI and TLI ranging from 0.94 to 0.98 and RMSEA ranging from 0.04 to 0.05. Minimal to slight improvement in model fit was found between M2 and M1 for the data from the U.S., South Korea, and England. Overall, the model M2 fits the data adequately across all four countries even though a slight reduction in model fit was observed for the data from Mexico. Table 5 summarizes the standardized factor loadings for the second-order CFA model. The factor loadings of the overall scale of teacher self-efficacy estimated on each of its three subscales across the four countries range from 0.70 to 0.85. Interested readers can contact the third author for the detailed visual figures of M1 and M2.

**Table 5:** Second-Order Confirmatory factor analysis (M2) results and measurement properties of the scales by country.

**Scale Items**	**Standardized loading**			
	**USA**	**Korea**	**England**	**Mexico**
**Teacher Professional Engagement (TPE)**	**α** **=** **0.85**	**α** **=** **0.82**	**α** **=** **0.86**	**α** **=** **0.60**
TT2G46A	0.836 (0.013)	0.841 (0.01)	0.835 (0.009)	0.460 (0.027)
TT2G46B	0.914 (0.008)	0.845 (0.009)	0.925 (0.006)	0.785 (0.021)
TT2G46D (recoded)	0.848 (0.014)	0.781 (0.011)	0.842 (0.01)	0.760 (0.022)
TT2G46F (recoded)	0.851 (0.011)	0.808 (0.01)	0.852 (0.008)	0.730 (0.019)
**Teacher Self-Efficacy (2nd Order Factor)**				
**Efficacy in Classroom Management**	**α** **=** **0.84**	**α** **=** **0.87**	**α** **=** **0.84**	**α** **=** **0.78**
	0.858 (0.027)	0.857 (0.008)	0.79 (0.02)	0.694 (0.019)
TT2G34D	0.638 (0.013)	0.925 (0.005)	0.852 (0.011)	0.762 (0.014)
TT2G34F	0.638 (0.035)	0.320 (0.02)	0.761 (0.028)	0.490 (0.026)
TT2G34H	0.900 (0.011)	0.905 (0.006)	0.884 (0.011)	0.859 (0.012)
TT2G34I	0.843 (0.016)	0.902 (0.006)	0.825 (0.014)	0.765 (0.014)
TT2G34E(cross-loading)	–	0.348 (0.022)	0.367 (0.025)	–
**Efficacy in Instruction**	**α** **=** **0.79**	**α** **=** **0.85**	**α** **=** **0.76**	**α** **=** **0.77**
	0.768 (0.026)	0.952 (0.008)	0.834 (0.02)	0.846 (0.015)
TT2G34L	0.851 (0.018)	0.856 (0.007)	0.852 (0.011)	0.791 (0.012)
TT2G34C	0.477 (0.036)	0.460 (0.029)	0.761 (0.028)	0.702 (0.015)
TT2G34J	0.785 (0.021)	0.861 (0.008)	0.884 (0.011)	0.743 (0.014)
TT2G34K	0.821 (0.017)	0.859 (0.008)	0.825 (0.014)	0.795 (0.013)
TT2G34G(cross-loading)	0.432 (0.031)	0.552 (0.025)	0.367 (0.025)	–
**Efficacy in Student Engagement**	**α** **=** **0.85**	**α** **=** **0.84**	**α** **=** **0.82**	**α** **=** **0.70**
	0.812 (0.024)	0.846 (0.009)	0.769 (0.019)	0.989 (0.015)
TT2G34A	0.929 (0.009)	0.905 (0.006)	0.907 (0.012)	0.660 (0.016)
TT2G34B	0.936 (0.009)	0.93 (0.005)	0.926 (0.01)	0.789 (0.012)
TT2G34E	0.825 (0.016)	0.57 (0.021)	0.511 (0.024)	0.592 (0.016)
TT2G34G	0.439 (0.032)	0.283 (0.025)	0.377 (0.032)	0.758 (0.014)
TT2G34C (cross-loading)	–	0.370 (0.029)	–	–
TT2G34F (cross-loading)	–	0.620 (0.019)	–	–
**Participation Among Stakeholders**	0.659 (0.025)^***^	0.761 (0.012)^***^	0.51 (0.033)^***^	0.79 (0.016)^***^
TT2G44A	0.816 (0.012)^***^	0.807 (0.008)^***^	0.848 (0.009)^***^	0.826 (0.018)^***^
TT2G44B	0.781 (0.014)^***^	0.754 (0.01)^***^	0.771 (0.011)^***^	0.784 (0.01)^***^
TT2G44C	0.771 (0.013)^***^	0.84 (0.009)^***^	0.774 (0.012)^***^	0.815 (0.008)^***^
TT2G44D	0.945 (0.007)^***^	0.915 (0.006)^***^	0.842 (0.013)^***^	0.903 (0.006)^***^
TT2G44E	0.883 (0.008)^***^	0.918 (0.006)^***^	0.797 (0.012)^***^	0.751 (0.015)^***^
TT2G45D (cross-loading)	–	0.265 (0.021)^***^	0.192 (0.023)^***^	0.388 (0.022)^***^
**Teacher-Student Relationship**	0.661 (0.025)^***^	0.787 (0.01)^***^	0.564 (0.029)^***^	0.73 (0.015)^***^
TT2G45A	0.818 (0.021)^***^	0.849 (0.011)^***^	0.883 (0.017)^***^	0.74 (0.017)^***^
TT2G45B	0.906 (0.012)^***^	0.876 (0.008)^***^	0.914 (0.011)^***^	0.846 (0.012)^***^
TT2G45C	0.898 (0.013)^***^	0.922 (0.008)^***^	0.889 (0.011)^***^	0.845 (0.012)^***^
TT2G45D	0.761 (0.017)^***^	0.492 (0.02)^***^	0.649 (0.018)^***^	0.375 (0.024)^***^
TT2G44D(cross-loading)	–	–	0.156 (0.025)^***^	−0.013 (0.026)^***^
TT2G44E(cross-loading)	–	–	0.223 (0.023)^***^	0.199 (0.021)^***^
**School Violence**	**α** **=** **0.74**	**α** **=** **0.77**	**α** **=** **0.73**	**α** **=** **0.82**
TC2G32D	0.654 (0.039)^***^	0.664 (0.018)^***^	0.415 (0.027)^***^	0.64 (0.016)^***^
TC2G32E	0.464 (0.041)^***^	0.827 (0.014)^***^	0.721 (0.015)^***^	0.868 (0.011)^***^
TC2G32F	0.58 (0.043)^***^	0.818 (0.015)^***^	0.878 (0.017)^***^	0.873 (0.011)^***^
TC2G32G	0.576 (0.034)^***^	0.406 (0.022)^***^	0.789 (0.017)^***^	0.774 (0.013)^***^

*α is reliability coefficient alphas. All loadings are significant at 0.001 level*.

*Note*:

*** *p < 0.001*;

** p < 0.01;

* *p < 0.05 (two-tailed); +p < 0.1*.

**Figure 2:**
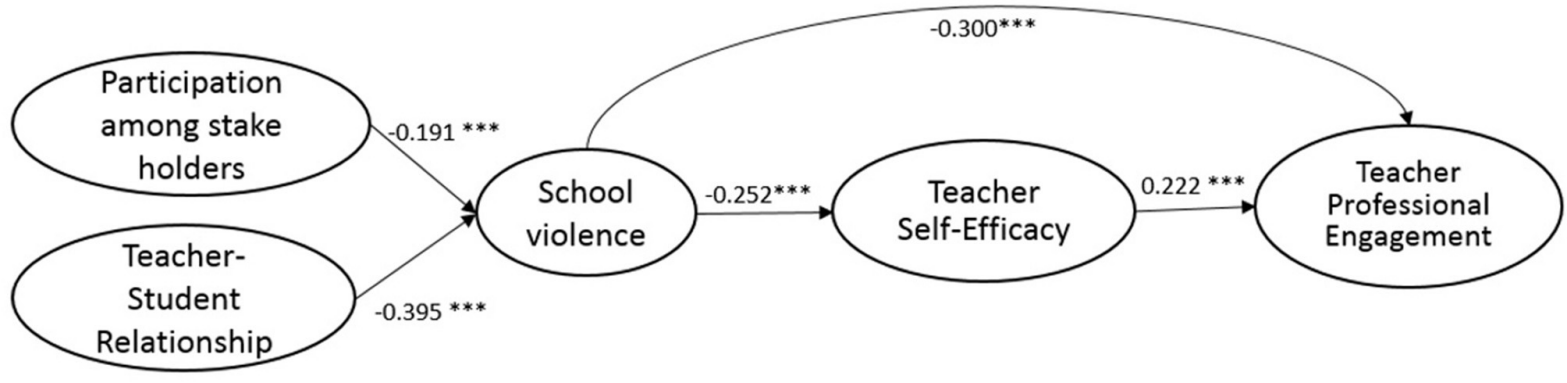
Standardized estimates of the SEM model (USA).

**Figure 3:**
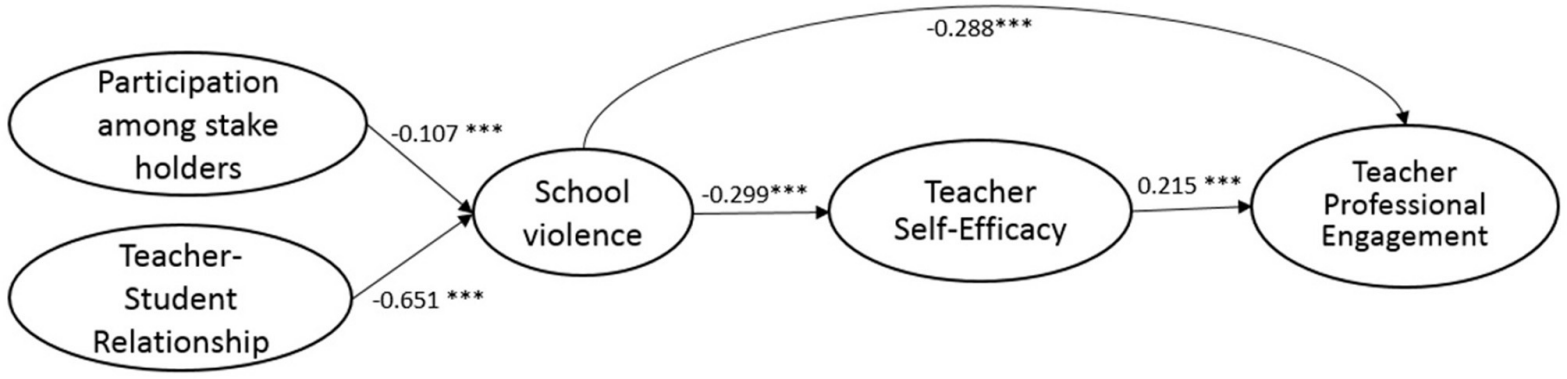
Standardized estimates of the SEM model (KOREA).

**Figure 4:**
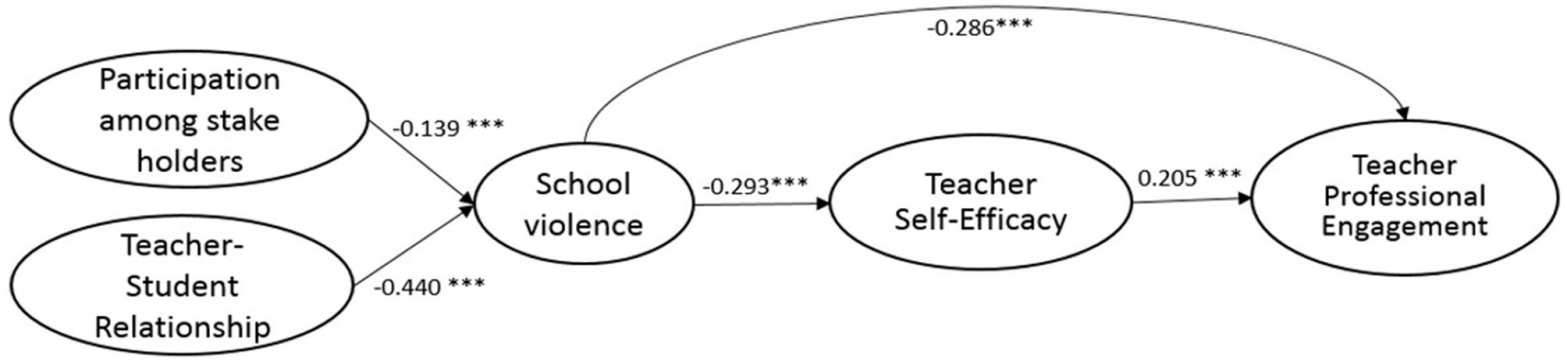
Standardized estimates of the SEM model (ENGLAND).

**Figure 5:**
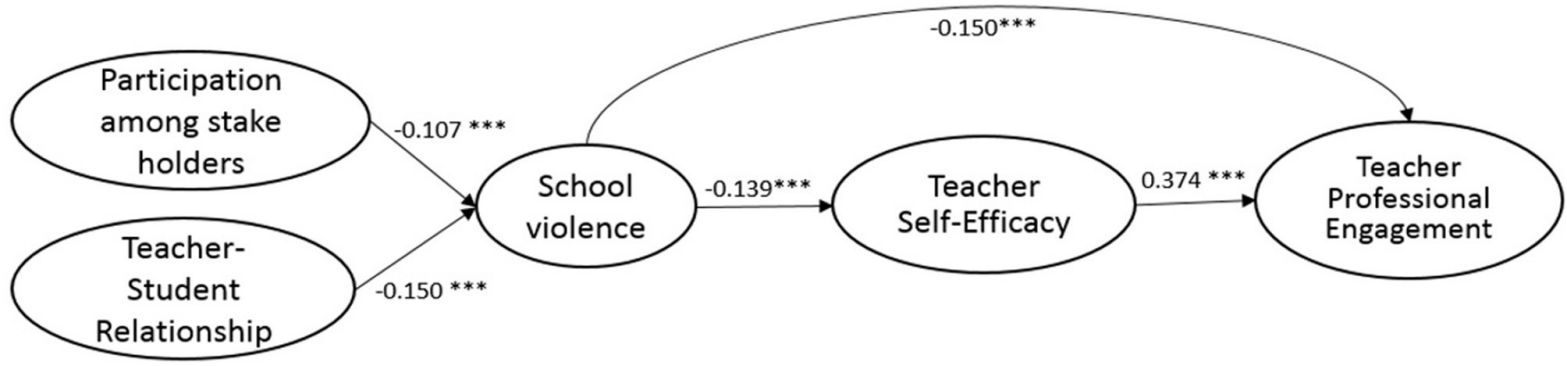
Standardized estimates of the SEM model (MEXICO).

The full SEM research model (M3) followed the same configural structure as M2 but added paths connecting the hypothesized relationship between the five constructs: participation among stakeholders, teacher–student relationship, school violence, teacher self-efficacy, and teacher professional engagement. Teachers' demographics and schools' context were added as control variables in M3. Though there is a slight reduction in terms of model fit compared to M2, the final hypothesized research model (M3) still shows adequate fit (see Table 4) for the samples from the U.S., South Korea, England, and Mexico. The standardized estimates of path coefficients and the effects of the control variables are presented in Table 6. The standardized estimates of path coefficients for the U.S., South Korea, England, and Mexico are also shown, respectively, in Figures 2–5.

**Table 6:** The effects of school violence on teachers professional engagement by country.

**Effect**	**Path**	**Standardized Estimate**			
		**USA**	**Korea**	**England**	**Mexico**
	Participation among stake holders -> School Violence	−0.191 (0.049)^***^	−0.107 (0.049)^***^	−0.139 (0.036)^***^	−0.107 (0.043)^***^
Direct Effect	Teacher–Student Relationship –> School Violence	−0.395 (0.049)^***^	−0.651 (0.042)^***^	−0.440 (0.036)^***^	−0.150 (0.028)^***^
	School Violence -> Teacher Self-Efficacy	−0.252 (0.044)^***^	−0.299 (0.026)^***^	−0.293 (0.031)^***^	−0.139 (0.032)^***^
	School Violence –> TPE	−0.300 (0.043)^***^	−0.288 (0.027)^***^	−0.286 (0.029)^***^	−0.150 (0.028)^***^
	Teacher Self-efficacy –> TPE	0.222 (0.038)^***^	0.215 (0.024)^***^	0.205 (0.028)^***^	0.374 (0.027)^***^
Indirect Effect	School Violence –> Teacher Self-Efficacy->TPE	−0.069 (0.016)^***^	−0.082 (0.011)^***^	−0.102 (0.017)^***^	−0.048 (0.010)^***^
Controls	Female	−0.030 (0.035)	0.016 (0.025)	0.033 (0.026)	0.085 (0.027)^**^
	Age	0.089 (0.039)^*^	−0.057 (0.025)^**^	−0.042 (0.033)	−0.017 (0.036)
	School-Related Work Experience	0.016 (0.039)	−0.062 (0.033)^+^	0.017 (0.031)	0.052 (0.036)
	Full Time	−0.041 (0.046)	0.014 (0.027)	0.066 (0.028)^*^	0.081 (0.028)^**^
	Public Schools	−0.064 (0.056)	−0.080 (0.033)^*^	−0.054 (0.026)^*^	−0.011 (0.030)
	Rural/Village	−0.084 (0.039)	−0.008 (0.037)	0.081 (0.038)^*^	0.036 (0.036)
	(Large) City	−0.032 (0.036)	−0.059 (0.035)^+^	0.002 (0.029)	−0.032 (0.032)
	School Enrollment	−0.098 (0.034)^**^	−0.030 (0.029)	0.009 (0.025)	0.036 (0.029)
	Over 30% Students from Socioeconomically Disadvantaged Homes	−0.037 (0.042)	−0.022 (0.042)	−0.027 (0.028)	0.011 (0.030)

*** *p < 0.001*;

** *p < 0.01*;

* *p < 0.05 (two-tailed)*;

+ *p < 0.1*.

Similar and consistent patterns were observed among the U.S., South Korea, England, and Mexico. *First*, significant and positive effects of teacher self-efficacy on teacher professional engagement (*teacher self-efficacy*→*teacher professional engagement*) were observed in the samples from the four countries. While the effects were similar among the samples of the U.S. (β = 0.222, *p* < 0.001), England (β = 0.205, *p* < 0.001), and South Korea (β = 0.215, *p* < 0.001), the effect was more prominent in the Mexico sample (β = 0.374, *p* < 0.001). *Second*, school violence had significant negative effects on teacher self-efficacy (*school violence*→*teacher self-efficacy*); the negative effects of school violence on teacher self-efficacy was the least profound in the Mexico sample (β = −0.139, *p* < 0.001), but were similar in extent among the samples of South Korea (β = −0.299, *p* < 0.001), England (β = −0.293, *p* < 0.001), and the U.S. (β = −0.252, *p* < 0.001). *Third*, participation among stakeholders had significant negative effects on school violence (*participation among stakeholders*→*school violence*), implying that an increase in participation among stakeholders can contribute significantly to the prevention of school violence. Among the four countries, the U.S. benefited the most from stakeholders' involvement (β = −0.191, *p* < 0.001), followed by England (β = −0.139, *p* < 0.001). Effects were found to be the same between South Korea (β = −0.107, *p* < 0.001) and Mexico (β = −0.107, *p* < 0.001). *Fourth*, good teacher–student relationships were found to have significant negative effects on the incidence of school violence (*teacher–student relationship*→*school violence*). This was observed in samples from all four countries, implying that positive teacher and student relationships can effectively reduce violence in schools. A closer look at the results revealed that the negative effects were most profound in the samples of South Korea (β = −0.651, *p* < 0.001), similar between the U.S. (β = −0.395, *p* < 0.001) and England (β = −0.440, *p* < 0.001), and the least profound in the Mexico sample (β = −0.150, *p* < 0.001). *Fifth*, significant and negative direct effects of school violence on teacher professional engagement (*school violence*→*teacher professional engagement*) were identified for all four samples. The extent of the effects was most adverse for the samples from the U.S. (β = −0.300, *p* < 0.001), followed by England (β = −0.286, *p* < 0.001), and South Korea (β = −0.288, *p* < 0.001), with the Mexico samples being the least adverse (β = −0.150, *p* < 0.001). *Sixth*, across all four countries, the indirect effects of school violence on teacher professional engagement through teacher self-efficacy were significant (see Table 6 for the estimates and *p*-values among the four countries). Slight differences in the proportion of the effect of school violence on teacher professional engagement, mediated through teacher self-efficacy, were found across the four samples with the U.S. being about 16%, England 17%, South Korea 18%, and Mexico 25%.

With regard to teachers' characteristics, the teacher's gender is not a significant predictor of their professional engagement except in the samples from Mexico, where female teachers responded as having a higher level of professional engagement (β = 0.027, *p* < 0.01). Teachers' age is positively correlated to their professional engagement (β = 0.089, *p* < 0.05) in the samples from the U.S., but negatively correlated in the samples from South Korea (β = −0.057, *p* < 0.01); no significant relationship between age and professional engagement was found in the samples from England and Mexico. Teachers' employment status shows that full-time teachers report higher professional engagement in samples from England (β = 0.066, *p* < 0.05) and Mexico (β = 0.081, *p* < 0.01) than part-time teachers do.

With respect to schools' contextual factors, teachers in public schools in South Korea (β = −0.08, *p* < 0.05) and England (β = −0.054, *p* < 0.05) reported significantly lower levels of professional engagement than teachers in private schools did. Schools' locations were found to be significant predictors only in England, where teachers in schools located in villages or rural areas reported significantly higher levels of professional engagement than teachers in schools in towns (β = 0.081, *p* < 0.05). School enrollment size is significant only in samples from the U.S., where increased enrollment resulted in less professional engagement as reported by teachers (β = −0.098, *p* < 0.01). No effect on professional engagement was associated with schools enrolling more or < 30% of students from socio-economically disadvantaged homes.

## Discussion

This study explored the impact of school violence on teacher professional engagement and how the impact may be alleviated by perceived participation among stakeholders, teacher–student relationships, and teacher self-efficacy among secondary school teachers from the U.S., England, South Korea, and Mexico. The four countries are from three different continents, each representing unique sociocultural values and educational systems. These countries were selected for inclusion in the study on the basis of the unique contribution each country could make to the understanding of school violence and teacher professional engagement. The utmost purpose of the study was to inform policy to reduce teacher turnover.

Structural equation modeling was employed to evaluate the conceptual model of the relationships. Aligning with previous studies, this study confirmed that school violence could have a significant and negative direct impact on a teacher's professional engagement (Janosz et al., 2004) and the negative impact can be alleviated and mediated by teachers' self-efficacy. The pattern is consistent across all four countries: a teacher's perception of insecurity and vulnerability due to violence at school negatively impacted their self-efficacy, which can lead to their reduced engagement in school. The direct impact of school violence on teacher professional engagement is slightly more adverse in the samples from the U.S. than in those from England or South Korea, and the impact is the least adverse in the samples from Mexico. In comparison to the other three countries, teacher self-efficacy in Mexico mediates a relatively higher proportion of the effect of school violence on teacher professional engagement. The different magnitudes of direct and indirect impact could be due to differences in the theoretical understanding and conceptualization of school violence that is unique to each country. A potential factor could be that teachers in Mexico tend to have a higher threshold of tolerance for some behaviors that have been classified as misdemeanors (Estévez et al., 2016). This study also reveals that, consistently, across all four countries, increased participation among stakeholders and positive teacher–student relationships plays an important role in reducing teachers' exposure to school violence. The findings of this study suggest that schools with higher levels of participation among stakeholders tend to have lower levels of school violence.

Parent engagement, community involvement, and the participation of other stakeholders have been integral components in conceptualizing the school climate (Moos, 1979; Cohen et al., 2009). More intensive research and clearer delineation of school climate models has granted schools and teachers in the U.S. and England a pragmatically better position to develop and implement initiatives involving parents and the community to foster a positive school climate, which also highlights the need for clearer conceptual models of school climate in Mexico (including countries in South America) and South Korea (as well as other Southeast Asian countries). The consensus agreement from the sampled teachers in the four countries is: to effectively address the problem of school violence and to deter students from conducting violent behaviors, collaboration and concerted efforts between the school, family, community, and other stakeholders are necessary.

Consistent with the previous literature, the findings in this study reveal that positive teacher and student relationships could foster emotional well-being and reduce the negative effects that school violence may have on teachers (Van Dick and Wagner, 2001). The results of the current study indicate that, comparatively speaking, positive teacher–student relationships have the most significant effect in reducing school violence in South Korea and the least profound effect in Mexico. Though vastly different in sociocultural contexts and geographical environments, South Korea and Mexico are somewhat similar in terms of how teachers are treated by students: as distant authority figures (they are often treated as peers in the U.S. and England). The highly valued hierarchy in South Korean culture leads to teachers having higher expectations about students being respectful and being less tolerant of student misbehavior. Perhaps the more respectful students are, the more harmonious the relationship is with their teachers, and the less likely these students are to commit violent acts in schools. However, research has suggested that a different type of teacher–student relationship is valued in schools in Mexico: the most successful students typically receive minimal attention from teachers, while only students of poorer academic achievement (or students with behavioral problems) feel connected to their teachers (Weiss and García, 2015).

According to theories of school climate, teacher–student relationships and participation and collaboration among stakeholders are among the defining dimensions of school climate (Moos, 1979; Cohen et al., 2009). The findings of our study have highlighted the significant bearing that the teacher–student relationship and collaboration among stakeholders have on the role of school climate in reducing school violence (Cohen and Freiberg, 2013; Bradshaw et al., 2015). Thus, even though the measures of violence prevention are rather broad, these results underscore positive student-teacher relationship building and proactive collaboration among stakeholders as the focus of school violence prevention across all four countries of different educational ideologies (Gottfredson and Gottfredson, 2001).

This study also shows that school violence yielded a significant negative impact on teachers' efficacy. Teachers from schools with higher levels of school violence tended to report lower teaching efficacy. These findings are consistent with studies showing that teachers reporting the lowest level of teaching efficacy are notably those who have experienced a lot of student misbehavior (Roberts et al., 2007). The negative impact of school violence on teacher self-efficacy was found to be consistent across the U.S., England, and South Korea, though to a lightly varying degree. The samples of teachers from Mexico reported less severe effects of school violence on their efficacy. A possible explanation could be that they conceptualize school violence and/or teachers' dismissiveness of problematic misdemeanor behaviors in school differently than teachers from the other three countries (Estévez et al., 2016).

Teachers spend most of their time in multiple intersecting contexts and their sense of wellbeing, self-efficacy, and teaching engagement are shaped by environmental factors. Extensive studies have indicated that the stability of teacher workforces has implications for educational quality and for child development (Ingersoll, 2001). Studying the extent to which school violence has impacted teachers' professional development can help us to better understand teacher turnover and attrition and can facilitate the development of retention policies. The findings of this study suggest that the negative emotional impact of school violence can be very influential to teacher turnover. Therefore, the prevention of school violence will not only benefit teacher well-being but will also help solve teacher shortages by enabling schools to retain more engaging teachers.

This study aimed at drawing more policy attention to cross-country studies in the field of school violence and teacher professional engagement. Nowadays, the demands on teachers are increasing around the world. Teachers are facing increasingly complex educational conditions (Sutcher et al., 2016). At the same time, the attractiveness of teaching as a profession in many countries is declining. It has been increasingly challenging for many countries and educational systems to recruit and retain highly qualified people (Qin, 2020). The results of this study indicate that school violence is prevalent across countries and teachers from different cultures and educational systems. A key factor in reducing school violence is to enhance teacher engagement.

In addition, the study findings also suggest that it is important to recognize, through international research, both the shared and unique norms and assumptions in terms of school violence and teacher professional engagement. Moreover, international collaborative efforts will help maximize the benefit of cross-national studies and minimize the potential consequences or missed opportunities that result from research and policy isolation.

## Conclusions and Limitations

The purpose of this study is two-fold. On the one hand, it aims to expand and deepen the understanding of relationships between school violence and teacher self-efficacy and professional engagement; on the other hand, it intends to help us better understand the extent to which school climate, in particular teacher–student relationships and collaboration between stakeholders and schools, contributed to the reduction of school violence. The vast majority of prior school violence studies have focused on the deleterious consequences of such violence on students. The current study has shown that school violence also affects teachers and could result in their professional disengagement. The results of this cross-national study suggest that the consequences of violence experienced by teachers should also be well-documented, especially because it is becoming a growing concern in many countries. The suggestion that educational policies should facilitate safer school environments for all students may not be sufficient and/or effective if the well-being of teachers has not been clearly and thoroughly addressed amidst efforts to develop school violence prevention strategies/plans.

Future research may need to focus on the impact of culture on teachers' perceptions of school violence. Future studies may also further address whether the links between school violence and teacher professional engagement are different in developed countries compared to developing countries. Finally, future research studies with robust datasets may identify more factors that contribute to school violence.

This research involved some limitations. For example, all the data from the TALIS database were self-reported by teachers and the school principals. Their self-enhancement biases may influence the objectivity of the responses (Alloy and Ahrens, 1987). Additionally, the measures of both exogenous and endogenous variables were obtained from the same TALIS cross-sectional survey; the shared variance may inflate correlations among variables of interest. Furthermore, instead of establishing a causal relationship between independent variables and teacher professional engagement, the intent of this study is to examine the nature and the degree of the relationships between the variables. Thus, any cause-and-effect implication remains uncertain. Moreover, as in all comparative studies, differences across countries may exist in various hard-to-observe ways. For instance, cultural traits, variation in school and educational management, and other characteristics associated with the variance of teacher professional engagement may all be significant. The unobserved heterogeneity between countries may increase the probability that the omitted variable caused bias in cross-national analyses. A related final limitation is that although we included four countries in this study, we did not conduct statistical multiple-group comparisons across the four countries; we stopped the testing of partial measurement invariance when a partial weak factorial invariance was established among the countries.

## Data Availability Statement

Publicly available datasets were analyzed in this study. This data can be found here: http://www.oecd.org/education/talis/talis-2013-data.htm.

## Author Contributions

LN performed the statistical analysis. All authors contributed to conception and design of the study, the manuscript drafting, revision, read, and approved the submitted version.

## Conflict of Interest

The authors declare that the research was conducted in the absence of any commercial or financial relationships that could be construed as a potential conflict of interest.
